# Returning home from a full-scale armed conflict: A rapid review of short post-deployment psychological practices

**DOI:** 10.1080/08995605.2025.2469329

**Published:** 2025-02-28

**Authors:** Tino Karolaakso, Kasperi Mikkonen, Tom Pakkanen, Petteri Simola, Kirsi Peltonen

**Affiliations:** a Faculty of Social Sciences, Tampere University, Tampere, Finland; b Psychiatry, University of Helsinki and Helsinki University Hospital, Helsinki, Finland; c Department of Psychology, Åbo Akademi University, Turku, Finland; d Finnish Defence Research Agency, Human Performance Division, Tuusula, Finland; e Invest Research Flagship Centre, University of Turku, Turku, Finland

**Keywords:** Service members, veterans, post-deployment, homecoming, mental health

## Abstract

After a full-scale armed conflict, tens of thousands of service members, including professional warfighters and reservists, return home facing an increased risk of PTSD and other mental disorders, as well as various reintegration difficulties that can impact their well-being, social relationships, and quality of life. Numerous countries and armed forces have developed post-deployment adaptation programs (PDAPs) and interventions to mitigate these risks. A rapid review was conducted to synthesize the research literature on rapid and short-term psychological support practices during the post-deployment homecoming phase of a full-scale armed conflict. The aim was to identify practices with scientific support when the homecoming phase is limited to a maximum of three days and that could be offered on a scalable basis to all returning service members. Several PDAPs and interventions were identified. The role of psychoeducation, help-seeking, and social support as other supportive practices was also assessed, suggesting possible interventions and online measures to increase these behaviors in the homecoming population.

## Introduction

Russia’s invasion of Ukraine in 2022 has set alight the largest full-scale conflict in Europe since World War II. A large portion of the nation’s available resources is utilized in the conflict. As the conflict subsides, a large number of service members transition to civilian life. These include both professional warfighters and reservists, whose background is conscript service, and most of them return to the civilian roles they left to join the war. The success of this transition is partly the responsibility of military organizations. Increased risks of mental disorders, including PTSD, as well as moral injury and civilian life problems after military members transition to civilian life are widely recognized (Rahnejat et al., 2022; Ramchand et al., 2015; Xue et al., 2015). While psychological support during deployment is crucial, the homecoming phase after deployment is also of high interest to stakeholders and national officials aiming to prevent the incidence of mental health disorders and behavioral problems in the veteran population.

The difficulties of transitioning and reintegrating into civilian life are explained by a complex interplay of psychological and social factors. In addition to mental disorders and moral injury, psychological factors primarily involve adjusting to various forms of loss, including the loss of military culture, community, identity, and a sense of purpose. The loss of purpose may also explain the challenges of integrating into civilian society (Ahern et al., 2015; Sachdev & Dixit, 2023). In the case of full-scale armed conflicts, however, it is important to recognize that the formation and nature of military identity can vary significantly depending on whether one is a voluntary professional warfighter or an activated reservist serving in national military service. Additionally, poor perceived social support during and after homecoming is associated with later PTSD and other mental health issues (Ramchand et al., 2015). Studies have also identified stigma among veterans regarding mental health disorders, with estimates indicating that 40–60% of veterans who would benefit from professional help never seek it (Hoge et al., 2004; Iversen et al., 2011; Kehle et al., 2010).

Numerous countries and armed forces have developed various post-deployment adaptation programs (PDAPs) and interventions to mitigate the risk of mental health and behavioral problems after deployment (Fertout et al., 2011; Hunt et al., 2014; Kennis & Brake, 2022). A substantial body of literature has evaluated the effectiveness of both holistic PDAPs and individual interventions in enhancing well-being, preventing PTSD and other mental health disorders, promoting help-seeking, and utilizing social support for service members transitioning home. Currently, however, research on support for homecoming of large military forces after full-scale conflicts is critically lacking.

As there is an urgent need for best practices in post-deployment support, addressing the identified gap in knowledge could be beneficial for various military organizations. Finland, for example, uses general compulsion for national military service with universal male conscription. The last full-scale conflict in Finland during World War II resulted in the army returning home without any post-deployment measures. These veterans suffered from a variety of mental and substance use problems. This not only affected them but also had a well-recorded impact on the health of their children and grandchildren (Ponteva, 1977). To mitigate the risks of postwar mental and substance use disorders and their intergenerational effects, gathering information and developing appropriate post-deployment psychological support measures for larger (conscript) armies is of utmost importance.

The current study aims to review the literature on feasible psychological support during the post-deployment phase after a full-scale armed conflict. This study expands upon a recent review that examined preventive support programs utilized throughout the different phases of deployment by focusing on the post-deployment phase (Cooper et al., 2024). Because of the lack of contemporary studies concerning support measures for larger military forces after full-scale armed conflicts, this review seeks to address this gap by hypothesizing and extrapolating existing support measures that could be adapted for the nearly simultaneous transition of tens of thousands of service members. Thus, the focus is forced to be on the existing evidence from smaller-scale conflicts and combat operations.

Moreover, transitioning entire armed forces into civilian life, especially under general conscription, presents significant logistical challenges, practical constraints, and requires many trained professionals. A homecoming phase that is too long is impractical, while one that is too short may be ineffective. Thus, this review assumes measures must be designed for implementation within a time frame of three consecutive days, aiming to ensure both feasibility and effectiveness during such a large-scale homecoming. Most existing research evaluates the impact of longer psychological practices and interventions requiring several weeks of regular attendance (e.g., Romaniuk & Kidd, 2018), which falls outside the scope of this review.

In addition to assessing the applicability and feasibility of interventions for tens of thousands of returning service members, we also discuss interventions aimed at a smaller population of a few hundred service members. This latter scenario aims to describe a detachment force sent to fight alongside a military alliance force in a foreign country. The research questions are: What are the rapid and short-term post-deployment psychological practices supporting the mental health of service members returning from a full-scale armed conflict? How can these practices be applied to i) a large population of tens of thousands of service members, and ii) a smaller population of hundreds of service members?

## Methods

To address the research questions, the authors conducted a rapid review to summarize the available evidence. The choice of the rapid review methodology was based on the need to address potentially immediate and emerging concerns in the context of large populations of service members returning after full-scale armed conflicts, as well as the current gaps in knowledge regarding scalable post-deployment practices. Rapid reviews are more vulnerable to error than systematic literature reviews because the search for studies is less comprehensive. The Cochrane recommendations for rapid reviews were used to evaluate the alignment between the current recommendations for rapid reviews and the conducted review (Garritty et al., 2024). Out of the 24 recommendations, this review fully met 16 criteria, partially met 2 criteria, and did not meet 6 criteria (Appendix 1). To analyze all possible evidence for applicable and feasible practices, all types of articles fitting the study question’s criteria were included.

The authors conducted several systematic searches. The main databases used were PubMed, the Cochrane Database of Systematic Reviews, Ovid Medline, APA PsycInfo and Scopus, with complementary searches with Google Scholar. The search queries were constructed based on combinations of the following search terms: “meta-analysis,” “review,” “military members,” “military populations,” “soldiers,” “post-deployment,” “homecoming,” “mental health,” “readjustment,” “reintegration,” “transition,” “adaptation,” “decompression,” and “community integration.” Separate searches were also carried out for journals specializing in military medicine (Military Psychology, Military Medicine, BMJ Military Health, Military Behavioural Health, Journal of Traumatic Stress), as well as from the reference lists of identified studies. The authors divided tasks by themes and conducted literature searches independently. All retrieved literature was available to all authors, and the articles included in the review were approved by all authors. The risk of bias and certainty of evidence analyses were omitted due to the heterogeneity of the included studies and their methodologies, as well as the broad scope of the review.

Inclusion criteria were: i) English-language, peer-reviewed articles or governmental armed forces reports; ii) studies published during the 21^st^ century, with priority given to meta-analyses, systematic reviews, and publications less than 10 years old; iii) the target population comprised post-deployment veterans or first responders in crisis situations. Exclusion criteria were: i) studies regarding only PDAPs, interventions, or practices that were not feasible within a maximum of three days for a large population of tens of thousands of service members, or a smaller population of hundreds of service members. All authors worked independently to extract the data relevant to the research questions from the included studies. A pre-determined extraction form was not used, the data was available to all researchers, and all questions and uncertainties related to the data were resolved collectively through discussion among all authors. The collected data were synthesized to present findings on identified general support practices and specific post-deployment programs and interventions.

## Results

The search produced 133 studies, from which 91 were excluded for meeting the exclusion criteria. The final sample consisted of 42 studies and reports, of which 16 studies covered PDAPs, 10 individual interventions, and 16 covered evidence for other support practices (e.g. facilitating help-seeking, social support). Of these, 5 were systematic reviews, 6 were other literature reviews, 8 were randomized controlled trials (RCTs), and 23 were other studies (e.g., non-RCTs, epidemiological studies, qualitative studies, feasibility studies). The flowchart of the study selection process is shown in Appendix 2. The included studies and their classifications are shown in Appendix 3. Appendix 4 details the feasibility of PDAPs and interventions identified as suitable for implementation within a maximum of a three-day post-deployment phase. For most practices, there is evidence advocating for their benefits within this time frame. Some have no published scientific studies on their effectiveness but are included and discussed based on other supporting literature related to risk and protective factors for veteran mental health. Results on the effectiveness of general support practices and specific interventions are presented.

### General practices to support the transition to civilian life

Supporting the transition from military to civilian life involves several key practices. Early preparation and planning for the transition are crucial for achieving better outcomes (Kleykamp et al., 2021). Given the constraints of the three-day post-deployment period, it is crucial to focus on psychoeducation and initiatives that lower barriers to seeking help and social support.

Psychoeducation aims to inform service members about the psychological effects that deployment circumstances and events may have after returning to civilian life. Psychoeducation can range from large-scale mass lectures attended by all returning service members, to guided small-group discussions. A review by Mulligan et al. (2011) found that while psychoeducation is associated with positive mental health outcomes and no adverse effects, the results are inconsistent, and the effect sizes are modest. Psychoeducation has been found to reduce negative mental health outcomes during the transition to civilian life, particularly when provided in interactive formats by a clinician and a military trainer. Psychoeducation is also highly valued by military personnel (Kennis & Brake, 2022).

Psychoeducation highlighting the daily changes, successes, difficulties, and coping mechanisms of civilian life can aid the transition process. Interventions aimed at reducing the likelihood of challenges and promoting adaptive coping strategies have been found beneficial (Larsson et al., 2024; Zamorski et al., 2012). For instance, self-guided dialogs about conflict experiences have been associated with positive emotional outcomes and greater social engagement (Milstein et al., 2022). Training in cognitive flexibility to manage common transition and reintegration challenges has been identified as potentially beneficial (Sachdev & Dixit, 2023; Zamorski et al., 2012). Psychoeducation on the temporary nature of problems, as well as the physiological and psychological effects of stress, is also recommended (Ahern et al., 2015; Zamorski et al., 2012).

Encouraging appropriate, timely, and independent help-seeking is crucial for maintaining functional capacity and positive mental health in a post-conflict population (Hoge et al., 2004; Iversen et al., 2011; Kehle et al., 2010). Barriers to seeking help include negative beliefs about its impact on one’s career (especially within the military context), fear of unequal treatment, being perceived as weak, negative beliefs about medication, preference for self-care, and practical challenges such as time constraints and geographical distance to services (Britt et al., 2016; Hoge et al., 2014). A qualitative study by Hinton et al. (2023) found that about three out of four veterans sought help in response to an acute crisis and often preferred treatments recommended by other veterans over those considered most effective by the latest research.

Veterans may have misconceptions about social norms, as a higher percentage personally approve of seeking help or say they would encourage others to seek help compared to what they believe others would approve of or do (Hitt & Massi Lindsey, 2020). Elevated treatment adherence has been observed in peer-assisted programs, highlighting the potential benefits of utilizing peers in lowering the threshold to seek help and normalizing the need for help (Hinton et al., 2023). Peer support can be facilitated through psychoeducational videos featuring veterans discussing their experiences and decisions to seek care (Garber & Zamorski, 2012; Mulligan et al., 2012).

A systematic literature review and meta-analysis by Xu et al. (2018) examined the efficacy of interventions aimed at increasing mental health-related help-seeking with a comprehensive data set of 98 studies (*N* = 69208). Its findings indicate that strategies to increase mental health literacy and to reduce stigma yield significant short-term benefits by increasing help-seeking behaviors and self-care. Interventions focusing on increasing motivation to seek help showed positive long-term effects on help-seeking.

### Post-deployment adaptation programs (PDAPs)

PDAPs usually consist of two elements: psychoeducation and decompression (Kennis & Brake, 2022). The key aspect of the decompression period is described as an opportunity to rest and relax with fellow service members. In a systematic literature review,Kennis and Brake (2022) assessed the structure, processes, and outcomes of PDAPs. Sixteen studies were included, from which they recognized two PDAP classes associated with positive mental health outcomes: Battlemind (BM) training and debriefing and Third Location Decompression (TLD). The quality was considered low for most studies, mainly due to the lack of a randomized design with an adequate control group, lack of baseline measurements, lack of controlling confounding factors, lack of description of intervention implementation, lack of use of a validated outcome measure, and lack of attention and analysis of dropouts.

In addition to BM and TLD, the Denning et al. (2014) report on programs aimed at preventing psychological disorders in service members highlights several post-deployment programs and interventions (e.g., Yellow Ribbon Reintegration Program and Wounded Warrior Programs), which we acknowledge as important efforts but which are outside the scope of this review.

#### Battlemind (BM) training and debriefing

BM is a training program used by the US Army and developed by the Walter Reed Army Institute of Research (Adler et al., 2009). BM training is designed to help service members adjust to life after deployment and deal with potential mental health challenges such as stress and PTSD. It is a holistic program providing support before, during, and after deployment (Adler et al., 2009; Mulligan et al., 2012). The BM training consists of six modules: three centered on pre-deployment for soldiers, leaders, and families; two address post-deployment adjustment; and the final module, for soldiers, is held 3–6 months after return. Currently, BM has been updated and rebranded as Deployment Cycle Resilience Training (DCRT) (Kirk et al., 2023). Consistent with the published literature, the program components for post-deployment support are referred to as BM training and BM debriefing.

In BM training, post-deployment problems are seen as the result of inappropriate psychological coping in a new situation. The aim is to reframe the problems into more positive and achievable challenges and to strengthen adaptive cognitions and coping skills. This includes strengthening skills and thinking patterns that help the individual to adapt and cope in the changed situation. BM includes an overview of effective and adaptive coping skills (e.g. stress and anger management, interpersonal conflict resolution and substance abuse prevention) and videos of service members transitioning home and/or with mental health problems (Adler et al., 2009; Garber & Zamorski, 2012; Mulligan et al., 2012; Zamorski et al., 2012).

In addition to the post-deployment training, the BM debriefing features an interactive group discussion where participants share difficult deployment experiences, with a particular focus on those related to transitioning back home. The debriefing addresses the service member’s transition to home as a critical psychosocial task and seeks to draw on the support of peers and group leaders. BM debriefing addresses criticism of traditional psychological debriefing by minimizing the reminiscence of past events. Instead, it emphasizes that these events occurred and are located in the past, and not part of the individual’s present experience (Adler et al., 2009; Kennis & Brake, 2022). Focusing as little as possible on the recollection of potentially traumatizing events, particularly on the detailed examination of their sensory memories, avoids re-traumatization and exposure of others to vicarious traumatization (Adler et al., 2009; McNally et al., 2003).

BM training and BM debriefing have been conducted by both military and civilian clinicians, as well as mixed teams including non-clinicians. The number of BM sessions varies from one to several sessions, with the number of participants ranging from two to 225 participants. The average duration of a single session is between 40 and 60 minutes (Kennis & Brake, 2022). Adler et al. (2009) investigated the use of BM training in both large groups (126–225 participants) and small groups (18–45 participants) in a comparative study (*N* = 2297 US soldiers returning from a one-year deployment in Iraq; 4-month follow-up with *N* = 1060). For PTSD symptoms, both BM debriefing as well as small- and large-group BM training resulted in lower symptoms compared to the active control (stress psychoeducation), but only in those with high levels of combat exposure. For depressive symptoms, BM debriefing resulted in lower symptoms compared to the control, but only in those with high levels of combat exposure. Large-group BM training resulted in lower symptoms compared to the control, regardless of combat exposure level. There were no significant differences in effects between small-group and large-group BM training.

A study by Castro et al. (2012) (*N* = 1645) evaluated the effects of 1 hour of BM training on US soldiers that had returned from Iraq four months earlier. In contrast to the original study, it was found that soldiers with both high and low levels of combat exposure had lower symptoms of PTSD and depression and higher life satisfaction at six-month follow-up. An immediate effect in reduced stigma associated with seeking mental health services was also reported, but this was no longer observed at the six-month follow-up.

In a study by Thomas et al. (2019) (*N* = 2439), small- and large group BM training showed no effects on PTSD and depressive symptoms compared to an active control (stress training) at 3-month follow-up. Soldiers in a small group rated the training more positively and reported higher levels of organizational support at follow-up.

Overall, the effect sizes of BM have been modest, ranging from small to medium. They are below 0.30 for individuals with high combat exposure and below 0.15 for those with low combat exposure (Adler et al., 2009; Castro et al., 2012; Thomas et al., 2019; Zamorski et al., 2012).

#### Third Location Decompression (TLD)

TLD is a decompression phase for returning service members that takes place in a third location rather than at home or in the deployment zone (Fertout et al., 2011). The purpose of TLD is to initiate and support the transition from the role of a service member toward civilian life. The decompression process allows disengagement from the stress, fears, and horrors of the battlefield, facilitating the readjustment to a civilian role and the normalization of various emotional experiences. A key aspect of TLD is to provide a relaxed, informal and safe environment which emphasizes peer support. TLD is used, for example, by the armed forces of the US, UK, Canada, and France following overseas deployments (Fertout et al., 2011; Wood et al., 2018). While many armed forces use a third country for TLD, the US has carried out the post-deployment period mainly in its own garrisons (Vermetten et al., 2014). Research has not indicated that a third country is necessary for TLD for health benefits (Castro, 2014).

Despite the widespread use of TLD, there is limited evidence on its benefits (Fertout et al., 2011). A study by Jones et al. (2011) with 11,304 British military personnel found that while about 80% were initially reluctant to participate in TLD, 91% found it useful afterward. Those most concerned about returning to civilian life reported the greatest benefits. The optimal duration for TLD also remains undetermined. British Armed Forces personnel advocate 36 hours as the most favorable length (Fertout et al., 2011; Kennis & Brake, 2022).

A study in the UK compared military personnel who participated in TLD with those who returned home directly (*N* = 3071) (Jones et al., 2013). The study sample was not randomized, but the researchers aimed to minimize bias through statistical methods. The study did not find differences in the ease of transition but noted that those with lower combat exposure experienced fewer PTSD symptoms, somatic symptoms and alcohol problems. These benefits were not observed in those with high combat exposure, possibly due to associated severe PTSD symptoms impairing their ability to benefit from TLD (Jones et al., 2013).

A quasi-experimental study by Schneider et al. (2016) found lower levels of PTSD and depressive symptoms in TLD participants compared to controls for six months post-transition to civilian life (*N* = 3143). The same difference was not observed across medical records. The authors suggested that TLD may have increased treatment-seeking among participants, leading to higher diagnosis rates than those who did not participate in TLD. While there are limited studies on the benefits of TLD, it is perceived positively by service members and has shown observable health benefits, which is why its continued use is recommended for those returning from deployment (Kennis & Brake, 2022).

### Post-deployment interventions

In addition to holistic PDAPs, individual interventions outside PDAPs have been studied in the context of transitioning service members. In their review, Bauer et al. (2018) evaluated the impact of various non-standardized interventions on the well-being of military personnel transitioning to civilian life. A wide range of interventions was identified: expressive writing, anger management, cognitive training, psychoeducation, relaxation techniques, resilience, and social cohesion. Most of these interventions require an extended period of time and multiple sessions to be implemented in the same way as they were studied. Among interventions implementable in a maximum of three days with demonstrated efficacy in that time frame are expressive writing (Baddeley & Pennebaker, 2011; Sayer et al., 2015), an Acceptance and Commitment Therapy (ACT)-based “Life Guard” workshop to promote resilience and reintegration (Blevins et al., 2011), and RESET training for managing and accepting intrusive thoughts (Shipherd et al., 2016).

#### Writing-based interventions

In an expressive writing intervention, a person is instructed to write about their deepest thoughts and feelings concerning a significant life event for up to 20 minutes a day for three to four days. This approach has been examined with service members, who were asked to write about their thoughts and feelings related to their transition to civilian life, either on paper or digitally, for 15–20 minutes per session. A study by Sayer et al. (2015) indicated that a short expressive writing intervention (average number of 2–3 daily sessions) reduced feelings of anger, perceived somatic problems, clinically significant anxiety, and PTSD likelihood, as well as increased employment rates at a six-month follow-up compared to non-writing peers (*N* = 1292). In a study by Baddeley and Pennebaker (2011), participants (average number sessions occurring every 10 minutes) reported higher relationship satisfaction at one month, but not at six months (*N* = 204).

Writing interventions are scalable and easy to implement, as instructions can be delivered to large groups, allowing veterans to continue independently after returning home. A low-resource intervention for home-returning service members involves initial short writing sessions with instructions for continued practice. This approach may be particularly beneficial for those without a diagnosable stress disorder, who have social support, and who have less combat experience (Frankfurt et al., 2019).

#### Workshops based on Acceptance and Commitment Therapy (ACT)

ACT is part of the third wave of cognitive behavioral therapy (CBT) aimed at enhancing psychological resilience through various psychosocial processes, including mindfulness training, defusing from and accepting one’s thoughts and feelings, identifying personal values, and committing to actions aligned with those values (Hayes et al., 2006). A growing body of literature supports the effectiveness of ACT alongside traditional CBT (Hayes & Hofmann, 2021). Studies on veteran populations have associated the practice of mindfulness with a lower prevalence of PTSD and depressive symptoms, reduced risk-taking behaviors, fewer pain symptoms and less functional impairment, as well as decreased self-stigma (Barr et al., 2022, Nassif et al., 2019).

Blevins et al. (2011) studied a two-hour ACT-based Life Guard (LG) workshop (*N* = 144). The workshop employs metaphors, acting, and role-playing (e.g., acting out a married couple on a journey and listening to their inner thoughts) facilitated by a team of clinical professionals, while avoiding traditional lecturing. Compared to their peers, those who completed the LG workshop reported fewer depressive symptoms and higher interpersonal satisfaction at two-month follow-up. Scaling this workshop for tens of thousands of service members could be highly challenging, but the model could be useful and feasible for volunteers, at-risk populations, or small groups of a few hundred individuals.

Similarly, in another study using the ACT framework, Shipherd et al. (2016) explored the effects of acceptance of involuntary and intrusive thoughts in soldiers who had recently returned from deployment. The one-hour RESET training (*N* = 1524) focuses on promoting acceptance and non-judgmental skills regarding thoughts and feelings to help participants deal with deployment-related intrusive thoughts, enabling them to observe their content without feeling as if the events were happening again. RESET training was associated with small but positive effects in reducing anxiety and functional impairment caused by intrusive thoughts compared to controls at a one-month follow-up. Control groups received training as usual (including BM), psychoeducation about intrusive automatic thoughts, and CONTROL training based on CBT, thus highlighting the benefits of the ACT-based workshop. Despite an RCT design, the study’s results may be biased due to the self-selection of participants and a high dropout rate (only 46% participated in the follow-up) (Bauer et al., 2018). Additionally, no significant differences were found between RESET and CONTROL training in reducing PTSD symptoms at follow-up (Shipherd et al., 2016).

#### Online interventions for help-seeking

Stand-alone digital platforms and short-term digital therapeutics may offer scalable solutions for reducing stigma and increasing help-seeking, which could be easily introduced to all service members transitioning to civilian life.

A study by Mengeling et al. (2024) examined the impact of Web-Ed on help-seeking among Reserve and National Guard service members returning from Iraq and Afghanistan (*N* = 414). Web-Ed includes symptom screening, psychoeducation, and links to Veterans Health Services materials (Mengeling et al., 2024). Easy access to post-deployment materials is crucial because changes in mental health can occur long after returning home (Hunt et al., 2014; Mengeling et al., 2024; Pietrzak et al., 2012). To the authors’ knowledge, there are no published RCTs of Web-Ed that assess its impact on help-seeking. However, studies show that over half of veterans who used Web-Ed reported gaining useful new information, and almost half planned to seek further treatment as a direct result of using Web-Ed (Mengeling et al., 2024).

A multimedia campaign known as Real Warriors (RW) has been used by the U.S. military to enhance the resilience, rehabilitation, and reintegration of veterans through various traditional and social media platforms, as well as dedicated websites (Denning et al., 2014). The RW campaign focuses on sharing service members’ stories about seeking help and the benefits they have gained, such as learning new coping skills and achieving success in their personal and professional lives. Studies on the RW campaign have shown positive results in increasing engagement and lowering the threshold for seeking help (Hong et al., 2021; Slay et al., 2021). Similar public awareness campaigns have generally had modest effects on increasing help-seeking behaviors (Denning et al., 2014). One potential approach to supporting a large population of service members returning home is to integrate these interventions with post-deployment media and web campaigns. This integration could reinforce the interventions’ content and taught skills while also serving as a reminder of available support resources.

#### Interventions aimed at increasing social support

Wu et al. (2012) implemented and studied the 512 Psychological Intervention Model (512 PIM) for Chinese military rescue workers in an RCT. This two-hour intervention (*N* = 2368), conducted about one month after an earthquake disaster with rescue workers as participants, combined Critical Incident Stress Debriefing (CISD) with group cohesion training, including teamwork games. The preventive effect of the model on PTSD and general mental health symptoms was not observed in the group receiving debriefing alone. This suggests that strengthening team cohesion plays a crucial role in reducing symptoms and increasing well-being post-disaster. A systematic review by Tan et al. (2022) examined the effects of interventions on PTSD and other mental health problems in military workers or first responders after a crisis event. They identified 512 PIM as the only intervention that reported preventing both symptoms of PTSD and general mental health problems.

Another scalable, social support-oriented approach is the guided dialogue method developed for the Warrior Spirit/Mission Homefront to support homecoming (Milstein et al., 2022). This gamified method facilitates discussion among service members about their experiences. Within a 90-minute intervention, service members (*N* = 299) used a specially designed deck of cards and a game to discuss and answer questions about themselves and their deployment. The deck contained 70 cards and 420 questions. The results of this feasibility study indicated high satisfaction among service members, with the method being associated with increased positive emotions and a greater willingness to talk with others (Milstein et al., 2022). The game and deck of cards are also recommended for use with close family and friends. No RCTs on the effects of this method have been published so far. Despite this, its scalability and acceptability suggest it may be a feasible way to encourage sharing of deployment experiences and seeking social support, even in large home-returning populations.

## Discussion

After a full-scale armed conflict, service members, both professional warfighters and activated reservists returning home, are at an increased risk of PTSD and other mental disorders and problems. This can affect their well-being, social relationships, and quality of life. This rapid review performs a synthesis of identified research literature on practices supporting the mental health of service members returning home. The focus was on practices that are feasible within a three-day time frame and for either tens of thousands of service members or a smaller population of hundreds of service members. Several PDAPs and interventions, as well as general supportive practices, were identified that have been studied through RCTs and subsequent systematic reviews (Bauer et al., 2018; Denning et al., 2014; Kennis & Brake, 2022; Tan et al., 2022; Xu et al., 2018).

In the case of a full-scale conflict with an army of tens of thousands of service members returning home, it is not feasible to implement individual interventions. The focus should be on facilitating the transition and reintegration into civilian life, providing psychoeducation, and preventing the development of mental and behavioral disorders and social problems. Earlier literature and established practices support the use of post-deployment TLD, which could take place in garrisons, holiday villages, or hotels capable of housing tens of thousands of service members while providing facilities for relaxation, informal activities, lectures, and workshops (Fertout et al., 2011; Jones et al., 2013; Kennis & Brake, 2022; Schneider et al., 2016). However, the mixed results regarding post-deployment TLD warrant more research.

In addition to rest and disengagement from the experiences of the conflict, BM training and debriefing are feasible and supported by the literature (Adler et al., 2009; Castro et al., 2012; Denning et al., 2014; Kennis & Brake, 2022; Thomas et al., 2019; Zamorski et al., 2012). Furthermore, several non-standardized interventions have shown feasibility, particularly writing- and ACT-based workshop interventions (Bauer et al., 2018). In addition to expressive writing, a systematic review and meta-analysis by Dawson et al. (2021) found that exposure-based writing therapy, which involves 15-minute writing sessions based on the Pennebaker paradigm, effectively reduced PTSD symptoms in nonmilitary members. Reduced PTSD symptoms were found in both sub-clinical and clinical samples, although one study found no difference compared to placebo writing (Dawson et al., 2021; Sloan et al., 2011). Exposure-based writing therapy (as a PTSD treatment modality) is also potentially highly scalable but typically requires professional support. No published studies on its potential adverse effects are known to the authors. This limitation should be considered a potential risk factor for the widespread self-administration of exposure-based writing therapy by service members transitioning to civilian life and is thus not included in the recommendations.

Studies on social support provide both direct (Wu et al., 2012) and primarily indirect (2017; Milstein et al., 2022; Tan et al., 2022) evidence for interventions aimed at increasing support-seeking behavior. The BM training program also addresses the stigma associated with seeking help for mental health problems and the barriers to seeking help (Adler et al., 2009; Zamorski et al., 2012). Online platforms can increase the reach of various practices, offering digital materials, guidance, and reminders of the coping skills taught before returning home (Denning et al., 2014; Mengeling et al., 2024). Although evidence for the effectiveness of non-standardized interventions is limited, a substantial body of research highlights these key aspects as crucial for enhancing veterans’ well-being. Therefore, high-quality research on this topic is urgently needed.

Overall, the quality of the research literature varies, and the effects observed in RCTs range from small to medium at best. However, even small effect sizes can translate into significant public health benefits, especially when a large percentage of the adult population have served in a major conflict. These effects can yield immediate positive outcomes for the post-full-scale conflict veteran population and potentially have cumulative intergenerational mental health benefits.

### Strengths, limitations, and future research

The strength of this rapid review is the inclusion of different kinds of research literature. The synthesis of different types of studies (e.g., systematic reviews, randomized controlled trials, qualitative research) provides a holistic approach to answering the research questions and creates opportunities for researchers to understand the wide range of themes related to post-deployment practices and to build upon them in further research. The limitations of this approach are that it is more prone to bias when evaluating the studies and synthesizing different types of scientific evidence. A rigorous evaluation of the risk of bias for these studies was not performed, as it would have required the use of several risk -of -bias tools. It is also possible that some studies fitting our inclusion criteria were not identified during the literature search, as many of the individual studies were over ten years old. Furthermore, the review did not include any gray literature, such as reports and policy briefs from various military organizations, which could have provided valuable insights for the research questions. Additionally, the search terms included soldiers but not other specific U.S. military populations, which may have limited the identification of practices tailored to specific subgroups of service members.

Another limitation is that the identified research mainly focuses on professional service members in overseas conflicts. To our knowledge, there is no research conducted on a large group of tens of thousands of service members returning home from a full-scale conflict (e.g., from the borders of one’s own country). Thus, the appropriate post-deployment practices are forced to be extrapolated based on the existing literature. Also, it is important to note that motivational factors and service member engagement in post-deployment measures are important regardless of which program or intervention is utilized. However, considering this with large homecoming armed forces and conscription armies was outside the scope of this study but is an important point for further research.

Given the somewhat contradictory results on the effectiveness of post-deployment practices and their modest effect sizes, the development of further iterations of PDAPs and interventions with higher-quality research is warranted. Future programs and interventions should be based on the latest understanding and models of biopsychosocial and cultural factors affecting post-deployment mental health. Different components of PDAPs could be further examined in RCTs to enhance understanding of what works and what does not. Also, RCTs for interventions facilitating social support- and help-seeking behaviors were not identified within the scope of this study. As social support and social cohesion are known to be critical factors in the successful transition and reintegration into civilian life, studies aiming to identify best practices for increasing these behaviors are warranted (Flack & Kite, 2021; Hermann et al., 2012; Wright et al., 2012).

## Conclusions

Early preparation and planning for post-conflict support practices are crucial in reducing the incidence of mental disorders and social problems in service members returning home while simultaneously minimizing future intergenerational effects. However, the quality of the research literature varies, and some preventative practices aimed at well-known risk factors lack empirical studies. This highlights significant gaps in knowledge and indicates the need for future high-quality research to establish best practices after a full-scale armed conflict.

## Supplementary Material

Supplemental Material

Supplemental Material

Supplemental Material

Supplemental Material

## Data Availability

The authors confirm that the data supporting the findings of this study are available within the article.
