# Supplementary material for: Returning home from a full-scale armed conflict: A rapid review of short post-deployment psychological practices
Source: Mil Psychol. 2025 Feb 28;38(2):199–210. doi: 10.1080/08995605.2025.2469329 (PMC12934161; doi:10.1080/08995605.2025.2469329)
Supplement: Supplemental Material [file HMLP_A_2469329_SM3133.docx]

Appendix 4. Post-deployment support practices after a full-scale armed conflict when the homecoming phase lasts a maximum of three days.

| Psychological post-deployment practice | Feasibility for tens of thousands of service members returning home | Feasibility for hundreds of service members returning home | Requirements |
| --- | --- | --- | --- |
| Third Location Decompression (TLD)  2-3 days  Includes group/team mutual rest and disengagement (e.g., Jones et al., 2011) | Feasible, but not in a third country. Recommended in environments better than deployment zones, allowing meaningful activities and relaxation among service members, with facilities for mass and group sessions. | Feasible. Recommended in environments better than deployment zones, allowing meaningful activities and relaxation among service members, with facilities for mass and group sessions. | Does not require specific expertise or education from the supporting staff but requires locations to house a high number of returning service members, ensuring safety and possibly moderate alcohol reintroduction. |
| Battlemind training  Approx. 1-2 hours  Includes psychoeducation and coaching on effective and adaptive coping skills, as well as video footage of military cases featuring soldiers in transition and/or with mental health problems (e.g. Adler et al., 2009). | Feasible in large groups up to nearly 300 service members. If psychoeducational video materials are utilized, they can be made readily available online for all service members returning home. | Feasible in large groups up to nearly 300 service members or small groups of 20-30 people. If psychoeducational video materials are utilized, they can be made readily available online for all service members returning home. | The supporting staff in various studies has been composed of a diverse mix of health care clinicians (military and/or civilian) and military personnel (including trained officers and military chaplains). All should have training concerning Battlemind training and its principles (approx. 6 hours). |
| Battlemind debriefing  Approx. 1 hour  Includes a group discussion with peers concerning experiences of returning home (e.g. Adler et al., 2009) | Feasibility depends on the number of supporting staff and facilities available. A maximum group size of around 30 people is recommended. Debriefing content is recommended to be carefully planned, with emphasis on homecoming, not on combat experiences. | Likely feasible. Debriefing content is recommended to be carefully planned, with emphasis on homecoming, not on combat experiences. | The supporting staff in various studies has been composed of a diverse mix of health care clinicians (military and/or civilian) and military personnel (including trained officers and military chaplains). All should have training concerning Battlemind debriefing (approx. 8 hours). |
| Expressive writing  Approx. 15-20 minutes at a time, either with short breaks or on consecutive days  Includes writing about the thoughts and feelings regarding returning home.  (Baddeley & Pennebaker, 2011; Sayer et al., 2015) | Feasible with scalable instructions and tools, including digital writing options. | Feasible with scalable instructions and tools.  With a smaller number of service members, integrating guided group discussions with expressive writing becomes feasible. | Instructions can be provided in verbal, paper or digital format, requiring no specific expertise. |
| Life Guard workshop  Approx. 2 hours  Includes metaphors, acting and role-playing, aiming to increase psychological resilience (Blevins et al., 2011) | The optimal number of participants is not known. Likely not feasible to implement with all returning service members. It may however be feasible for a smaller group of volunteers or those at risk for developing a mental health disorder. | Likely feasible for all, when the number of service members returning home are in hundreds. | The supporting staff consisted of a psychologist, a social worker, a nurse and a recreational therapist in the original study.  Requires a sufficient basic understanding of Acceptance and Commitment Therapy (ACT). |
| Acceptance-based RESET training  Approx. 1 hour  Includes training in acceptance and non-judgemental skills in dealing with intrusive thoughts (Shipherd et al., 2016) | The optimal number of participants is not known. Likely not feasible to implement with all returning service members. It may however be feasible for a smaller group of volunteers or those at risk for developing a mental health disorder. | Likely feasible for all when the number of service members returning home are in hundreds. | The supporting staff was consisted of a doctoral-level counseling psychologist, and two master’s-level trainers. Requires a sufficient basic understanding of ACT. |
| Workshop for the familiarization of a platform for psychoeducation, self-care material and digital symptom monitoring platform  Cf. Web-Ed and Real Warriors campaign (Denning et al., 2014; Mengeling et al., 2024) | Feasible as an online digital platform for all service members. Implementation could also be supported with the existing digital mental health resources already in place in many countries. Presentation of platform is also possible in mass format. However, no RCTs were identified regarding benefits, but also the expected risks are low compared to the potential benefits. | Feasible as an online digital platform for all service members. Implementation could also be supported with the existing digital mental health resources already in place in many countries. Presentation of platform is also possible in mass format. However, no RCTs were identified regarding benefits, but also the expected risks are low compared to the potential benefits. | Staff should familiarize with platform/material. Expertise on mental health is likely to be useful, but not necessary. Potential benefits in including military peers to present the platform. |
| 512 Psychological Intervention  Model  Approx. 2 hours  Includes a CISD-model debriefing and a group cohesion training component (Wu et al., 2012) | Ten participants on average. Likely not feasible to implement with all returning service members. It may however be feasible for a smaller group of volunteers or those at risk for developing a mental health disorder.  Instead of a CISD-model debriefing, a Battlemind-model debriefing might be supported. | Likely feasible for all, when the number of service members returning home are in hundreds. | The supporting staff was consisted of clinical psychologists with two days of training in delivering the intervention. One psychologist was responsible for delivering the intervention to one group. |
| The Warrior Spirit/Mission Homefront gamified guided dialogue method  Approx. 1,5 hours  Includes a specially designed deck of cards and a game to facilitate discussions concerning the service members and their deployment experiences  (Milstein et al., 2022) | Feasible with scalable instructions and tools, also to support social interactions with the service members’ family and friends at home.  However, no RCTs were identified regarding benefits, but also the expected risks are low compared to the potential benefits. | Feasible with scalable instructions and tools. With a smaller number of service members, integrating guided group discussions becomes feasible. | Instructions can be provided in verbal, paper or digital format, requiring no specific expertise. |
