# Supplementary material for: Returning home from a full-scale armed conflict: A rapid review of short post-deployment psychological practices
Source: Mil Psychol. 2025 Feb 28;38(2):199–210. doi: 10.1080/08995605.2025.2469329 (PMC12934161; doi:10.1080/08995605.2025.2469329)
Supplement: Supplemental Material [file HMLP_A_2469329_SM3130.docx]

Appendix 3. The identified studies and their classification.

| Post-deployment adaptation programs (PDAPs) | Individual interventions | General support practices | Systematic reviews | Other reviews | Randomized controlled trials (RCTs) | Other studies (e.g. non-RCTs, epidemiological, qualitative studies, feasibility studies) |
| --- | --- | --- | --- | --- | --- | --- |
| Adler et al., 2009; Castro et al., 2012; Castro, 2014; Denning et al., 2014; Fertout et al., 2011; Garber & Zamorski, 2012; Jones et al., 2011, 2013; Kennis & te Brake, 2022; McNally et al., 2003; Mulligan et al., 2012; Schneider et al., 2016; Thomas et al., 2019; Vermetten et al., 2014; Wood et al., 2018; Zamorski et al., 2012 | Baddeley & Pennebaker, 2011; Bauer et al., 2018; Blevins et al., 2011; Frankfurt et al., 2019; Mengeling et al., 2024; Milstein et al., 2022; Sayer et al., 2015; Shipherd et al., 2016; Tan et al., 2022; Wu et al., 2012 | Ahern et al., 2015; Britt et al., 2016; Flack & Kite, 2021; Hermann et al., 2012; Hinton et al., 2023; Hitt & Massi Lindsey, 2020; Hoge et al., 2004, 2014; Hong et al., 2021; Iversen et al., 2011; Kehle et al., 2010; Kleykamp et al., 2021; Larsson et al., 2024; Mulligan et al., 2011; Sachdev & Dixit, 2023; Slay et al., 2021; Wright et al., 2012; Xu et al., 2018; | Bauer et al., 2018; Denning et al., 2014; Kennis & te Brake, 2022; Tan et al., 2022; Xu et al., 2018 | Castro, 2014; Fertout et al., 2011; McNally et al., 2003; Mulligan et al., 2011; Sachdev & Dixit, 2023; Vermetten et al., 2014; | Adler et al., 2009; Baddeley & Pennebaker, 2011; Castro et al., 2012; Mulligan et al., 2012; Sayer et al., 2015; Shipherd et al., 2016; Thomas et al., 2019; Wu et al., 2012 | Ahern et al., 2015; Blevins et al., 2011; Britt et al., 2016; Flack & Kite, 2021; Frankfurt et al., 2019; Garber & Zamorski, 2012; Hermann et al., 2012; Hinton et al., 2023; Hitt & Massi Lindsey, 2020; Hoge et al., 2004, 2014; Hong et al., 2021; Iversen et al., 2011; Jones et al., 2011, 2013; Kehle et al., 2010; Kleykamp et al., 2021; Larsson et al., 2024; Mengeling et al., 2024; Milstein et al., 2022; Schneider et al., 2016; Slay et al., 2021; Wood et al., 2018; Wright et al., 2012; Zamorski et al., 2012 |
