# Supplementary material for: Returning home from a full-scale armed conflict: A rapid review of short post-deployment psychological practices
Source: Mil Psychol. 2025 Feb 28;38(2):199–210. doi: 10.1080/08995605.2025.2469329 (PMC12934161; doi:10.1080/08995605.2025.2469329)
Supplement: Supplemental Material [file HMLP_A_2469329_SM3129.docx]

Appendix 2. Flowchart of study selection. The bidirectional arrow indicates that both boxes contain the same studies, categorized differently.

**Inclusion:** 133 studies

16 studies on post-deployment adaptation programs (PDAPs)

­­­­­­­­­­­10 studies on individual interventions

16 studies with only indirect evidence, mainly for general support measures

**Exclusion:** 91 studies due to the reported practices not being feasible in a maximum of three days for either a large population of tens of thousands of soldiers, or a smaller population of hundreds of soldiers

Final sample: 42 studies

5 systematic reviews

­­­­­­­6 other types of literature reviews

8 randomized controlled trials (RCTs)

23 other studies (e.g. non-RCTs, epidemiological studies, qualitative studies, feasibility studies)
