# Supplementary material for: Returning home from a full-scale armed conflict: A rapid review of short post-deployment psychological practices
Source: Mil Psychol. 2025 Feb 28;38(2):199–210. doi: 10.1080/08995605.2025.2469329 (PMC12934161; doi:10.1080/08995605.2025.2469329)
Supplement: Supplemental Material [file HMLP_A_2469329_SM3126.docx]

Appendix 1. Alignment between the Cochrane recommendations for rapid reviews and this study.

After reviewing the Cochrane recommendations, we conclude that, out of the 24 recommendations, we fully meet 16 criteria, partially meet 2 criteria, and do not meet 6 criteria.

- 1. We involved potential knowledge users to refine the review question, eligibility criteria, and outcomes of interest, with consultation at various stages of the review. The review was done in collaboration with a researcher affiliated with Defence Reseach Agency of the authors’ country of origin (MET CRITERIA).
- 2. We developed and reported our protocol which includes the review questions, population, interventions, comparators, outcomes, and methods of conducting the review (MET CRITERIA, described in the ‘Methods’ section of our manuscript).
- 3. The following limitations were in place when conducting the review: limiting the setting to involve scalable and feasible interventions and practices within a specific time-frame in the context of large military member populations, limiting the publication language to English (MET CRITERIA, described in the ‘Methods’ section, justification of the limitation is provided in the ‘Introduction’).
- 4. The authors include experts with experience both in the subject-matter of the review and in conducting systematic reviews (MET CRITERIA)
- 5. Multiple databases with additional searches from specific journals were used when conducting the review. The databases included Cochrane-recommended databases for both randomized and non-randomized studies (MET CRITERIA, the databases are described in ‘Methods’).
- 6. The search strategies were double checked for typographical errors, missed key words, and overall structure (MET CRITERIA).
- 7. We assess the need for grey literature (MET CRITERIA, an addition was made under ‘Strengths, Limitations, and Future Research).
- 8. and 9. the study selection was conducted by all authors (with specific roles related to selected topics) and worked individually and not blinded to one another. This was done to save time and to be able to cover multiple relevant themes (e.g. general practices, PDAP’s and post-deployment interventions) within one review (PARTIALLY MET CRITERIA, this process was added under ‘Methods’.
- 10. The data extraction was limited to only the most important data fields relevant to address the review question (MET CRITERIA, the data extraction was done manually and guided by the research questions).
- 11. A separate piloting exercise to allow team members to test data extraction was not conducted due to the different nature of the data extraction within each topic covered by the review (PARTIALLY MET CRITERIA/NOT APPLICABLE)
- 12. Data extractions relevant to conclusions were verified by multiple authors (MET CRITERIA).
- 13. Data extractions was done from both systematic reviews and primary studies based on the context (MET CRITERIA, e.g. Tan et al., 2022 for systematic review and Sayer et al., 2015 for primary study).
- 14-16. Risk of bias assessment was not made in the context of this review. (DID NOT MEET CRITERIA).
- 17. A descriptive summary of the included studies is presented in the review (MET CRITERIA, the summaries are provided under each theme and in Appendix 3).
- 18. A synthesis of the findings is presented in the review (MET CRITERIA, a synthesis is provided in ‘Discussion’.)
- 19. A meta-analysis was not appropriate in the scope of this review (MET CRITERIA/NOT APPLICABLE).
- 20. We focused on available systematic reviews and meta-analysis when conducting the review and strived to select primary studies based on their quality and relevance to the research question. An emphasis is put on systematic reviews and meta-analyses when drawing conclusions (PARTIALLY MET CRITERIA).
- 21-23. GRADE approach was not used to assess certainty of evidence (DID NOT MEET CRITERIA)
- 24. We provide a clear description of the selected review approach, which includes outlining the restricted methods used. Additionally, discuss the potential limitations of these chosen methods and how they may influence the interpretation of the research findings (MET CRITERIA).
